# Predictors of survival in critically ill patients with acute respiratory distress syndrome (ARDS): an observational study

**DOI:** 10.1186/s12871-016-0272-4

**Published:** 2016-11-08

**Authors:** Felix Balzer, Mario Menk, Jannis Ziegler, Christian Pille, Klaus-Dieter Wernecke, Claudia Spies, Maren Schmidt, Steffen Weber-Carstens, Maria Deja

**Affiliations:** 1 Department of Anesthesiology and Intensive Care Medicine, Charité - Universitätsmedizin Berlin, Campus Virchow-Klinikum / Campus Charité Mitte, Augustenburger Platz 1, D-13353 Berlin, Germany; 2 Department of Anesthesiology and Intensive Care Medicine, Charité - Universitätsmedizin Berlin, Campus Benjamin Franklin, Hindenburgdamm 30, 12203 Berlin, Germany; 3 SOSTANA GmbH, Wildensteiner Str. 27, 10318 Berlin, Germany; 4 Department of Anaesthesiology and Intensive Care Medicine, Werner Forßmann Krankenhaus, 16225 Eberswalde, Germany

**Keywords:** Acute respiratory distress syndrome, Pao2/FIO2 ratio, Oxygenation index, Classification, Risk stratification, Outcome

## Abstract

**Background:**

Currently there is no ARDS definition or classification system that allows optimal prediction of mortality in ARDS patients. This study aimed to examine the predictive values of the AECC and Berlin definitions, as well as clinical and respiratory parameters obtained at onset of ARDS and in the course of the first seven consecutive days.

**Methods:**

The observational study was conducted at a 14-bed intensive care unit specialized on treatment of ARDS. Predictive validity of the AECC and Berlin definitions as well as P_a_O_2_/F_i_O_2_ and F_i_O_2_/P_a_O_2_*P_mean_ (oxygenation index) on mortality of ARDS patients was assessed and statistically compared.

**Results:**

Four hundred forty two critically-ill patients admitted for ARDS were analysed. Multivariate Cox regression indicated that the oxygenation index was the most accurate parameter for mortality prediction. The third day after ARDS criteria were met at our hospital was found to represent the best compromise between earliness and accuracy of prognosis of mortality regarding the time of assessment. An oxygenation index of 15 or greater was associated with higher mortality, longer length of stay in ICU and hospital and longer duration of mechanical ventilation. In addition, non-survivors had a significantly longer length of stay and duration of mechanical ventilation in referring hospitals before admitted to the national reference centre than survivors.

**Conclusions:**

The oxygenation index is suggested to be the most suitable parameter to predict mortality in ARDS, preferably assessed on day 3 after admission to a specialized centre. Patients might benefit when transferred to specialized ICU centres as soon as possible for further treatment.

**Electronic supplementary material:**

The online version of this article (doi:10.1186/s12871-016-0272-4) contains supplementary material, which is available to authorized users.

## Background

The American-European Consensus Conference (AECC) definition was commonly used by clinicians to categorize ARDS patients [1]. But issues regarding reliability of various criteria have emerged, including a poor interobserver reliability of chest radiograph interpretation, confusing acute lung injury (ALI) /ARDS nomenclature and the inconsistency of PaO2/FiO2 ratio due to the effect of positive end-expiratory pressure (PEEP) [2]. Those limitations have recently been tackled with the establishment of the Berlin Definition in 2012 [3]. Herein, ARDS patients are classified into three independent categories (i.e., mild, moderate and severe ARDS) and additional variables are taken into account. Several factors, such as severity of chest radiograph, a PEEP level above 5 cm H_2_O, low compliance and poor oxygenation, are now used to define severe ARDS.

The Berlin Definition addresses and clarifies some of the limitations of the AECC definition and is the first to include minimum ventilator settings. The predictive validity for mortality is only slightly better than in the AECC definition. However, it was not designed to serve as a prognostication tool [2]. In the past, other variables of interest, such as the PaO2/FiO2 ratio, the oxygenation index (OI), the influence of co-morbidities and a number of clinical scores (SAPS, SOFA), were screened in ARDS patients to prompt early prediction of outcome and to ensure more efficient resource allocation [4–6]. As the ARDS is, however, a very heterogeneous syndrome with several different causes, all proposed definitions, parameters and variables did not resolve the problem adequately. For a reliable prediction of outcome and mortality in ARDS patients, comparable treatment strategies in respective hospitals are required. However, treatment algorithms applied on ARDS patients may differ significantly between different hospitals (usage of inhalative nitric oxide, ventilator settings, criteria to start lung assist devices) representing different approaches to the “state of the art” in ARDS therapy. These problems may be partly resolved in unicentric studies in specialized hospitals involving sufficiently high numbers of patients.

Outcome prediction in critically ill patients at a given point of time plays a major role (e.g. for appropriate treatment decisions and family communication). In this context, we examined the predictive values of the AECC and Berlin definition and also assessed alternative clinical parameters that are available in routine patient care.

## Methods

This observational analysis was conducted at a 14-bed intensive care unit (ICU) of a national reference centre specialized on treatment of ARDS in adult patients with severely compromised medical conditions. On average, two thirds of all ARDS patients are transferred from other hospitals. Patients at our institution were treated according to a strong treatment algorithm [7, 8].

After written consent of the Ethics Commission at Charité - Universitätsmedizin Berlin (EA1/223/12), clinical routine data from all patients admitted for ARDS between January 2007 and December 2013 were extracted from the two electronic patient data management systems operated at the hospital (COPRA, Sasbachwalden, Germany and SAP, Walldorf, Germany). In addition to basic demographic data, we assessed length of stay and duration of prior mechanical ventilation in referring institutions, comorbidities (using Charlson comorbidity score [9]), ICU admission scores, and use of extracorporeal oxygenation in order to characterize the patient population. As major clinical causes leading to ARDS, we differentiated pneumonia, sepsis of extra-pulmonary origin, trauma, immunedeficiency and “acute on chronic”, i.e. patients with an acute pulmonary disease on pre-existing chronic pulmonary disease (e.g.primary lung fibrosis, COPD >/= GOLD 4 or cystic fibrosis), because these are well known influencing factors of mortality in ARDS patients [10, 11].

Day 1 of study inclusion was defined as the first day with a median P_a_O_2_/F_i_O_2_ below 300 at our hospital. Patient-specific data that was extracted on a daily basis comprised SOFA score, ventilator settings / respiratory parameters (tidal volume (V_T_), tidal volume / predicted body weight (V_T_/PBW), P_mean_, P_peak_, PEEP, static compliance, F_i_O_2_), gas exchange using arterial blood gas analyses (pH, P_a_O_2_, P_a_CO_2_, P_a_O_2_/F_i_O_2_), use of nitric oxide and positioning therapy. Status of ARDS was assessed according to the definition of AECC [1] and the Berlin definition [3] upon admission on our ICU.

For analysing data based on ventilator settings and arterial blood gas analyses, the following algorithm was applied: Each day was divided in four intervals of six hours each. In each interval, the combination of ventilator settings and results of blood gas analyses with the least difference in time was chosen. For each of these parameters, the median was calculated and transferred to the study database. Ventilator settings had been saved approximately every 30 min in the electronic patient records and were only considered when they were documented prior to lab results.

Predictive validity for the AECC and Berlin definition as well as for P_a_O_2_/F_i_O_2_ and F_i_O_2_/P_a_O_2_*P_mean_ (OI) regarding mortality was assessed with receiver operator curves (ROC) and corresponding results for area under the curve (AUC). Kaplan-Meier curves were used to illustrate differences in survival using these four mentioned parameters. In order to show differences for continuous variables (i.e. P_a_O_2_/F_i_O_2_ and F_i_O_2_/P_a_O_2_*P_mean_), we selected the value that maximized the vertical distance between ROC curve and diagonal line (highest sum of sensitivity and specificity) [12]. This cut-off value was used to attribute patients to one of two groups (i.e. above or below calculated cut-off) in order to analyse predictive validity.

Descriptive analyses and statistical testing were performed using the R Project of Statistical Computing 3.0.1 with a *p* value below 0.05 regarded as significant. When normal distribution was ruled out using the Kolmogorov-Smirnov test, results were given in median and interquartile range (IQR), otherwise mean ± standard deviation (SD). Qualitative observations were characterized by numbers with percentage. Statistical significance among groups was univariately analyzed by the exact nonparametric Kruskal-Wallis-test and (pairwise) with the exact Mann–Whitney *U* test. Exact Chi-Square tests were used for qualitative data. In order to test multivariately for influencing factors of mortality and survival, Cox regression was applied with stepwise backwards selection including variables that showed a statistically significant impact in univariate analyses. All tests should be understood as constituting explorative analysis, such as no adjustment for multiple testing has been made.

## Results

The population analysed in this study comprised 442 critically-ill patients admitted for ARDS. As reflected by a median APACHE II admission score of 28 [20;35], a SAPS II admission score of 54 [39;70], a SOFA admission score of 12 [9;15] and a Charlson comorbidity index (CCI) of 3 [2–5], the study population was characterised by severe medical conditions (see Table 1). Along the line, patients required in median a PEEP of 17 [15;20] cmH_2_Obar, P_mean_ of 25 [21;29] cmH_2_O, and P_peak_ of 36 [32;39] cmH_2_O on day 1 of protocol. Further respiratory parameters are shown in Additional file 1: Table S1 in the electronic supplement, indicating more invasive ventilation in non-survivors. In 89.3 % of all patients, prone position was applied at least once within the first three days of protocol (see Additional file 2: Table S3 for further details). Non-survivors were significantly older than survivors. Also, lengths of stay in referring institutions as well as lengths of prior mechanical ventilation were longer in non-survivors. Although the aetiology of ARDS was overall not statistically different between survivors and non-survivors, the number of patients with acute on chronic respiratory failure was higher in non-survivors; trauma and sepsis of extrapulmonary origin were more frequent in survivors. Scores for description of severity of illness on ICU admission at our centre were significantly higher in the group of non-survivors. Extracorporeal lung assist devices (ELAD) including extracorporeal membrane oxygenation (ECMO) and extracorporeal lung assist (ECLA) were applied in 256 patients (57.9 %). When ELAD was required in patient, it was set up within the first three days of protocol in 87.5 % (see Additional file 3: Table S4 for further details). Overall patient survival with ELAD was 43.0 %.

**Table 1:** Patient characteristics and comparison between survivors and non-survivors at diagnosis of acute respiratory distress syndrome

	All patients	Survivor	Non-survivor	*p* - value
	*n* = 442	*n* = 240	*n* = 202	
Basic characteristics				
Age [years]	50.0 (37.0;61.0)	46.0 (34.0;60.0)	53.0 (42.0;64.0)	0.001*
Sex (male)	285 (64.5 %)	158 (55.4 %)	127 (44.6 %)	0.602
Weight [kg]	80.0 (70.0;95.0)	83.0 (70.0;100)	80.0 (69.5;90.0)	0.001*
Body mass index [kg/m^2^]	28.4 (8.46)	29.6 (8.81)	26.8 (7.73)	0.001*
Transfer from other hospital	307 (69.5 %)	165 (53.7 %)	142 (46.3 %)	0.878
LOS hospital before admission [d]	7.0 (3.0;15.0)	6.0 (3.0;10.2)	11.0 (4.0;23.0)	<0.001*
LOS ICU before admission [d]	4.0 (2.0;11.0)	4.0 (2.0;7.0)	6.0 (2.0;13.5)	0.002*
Duration of mechanical ventilation before admission [d]	3.0 (2.0;7.0)	3.0 (2.0;6.0)	4.0 (2.0;11.0)	0.002*
Severity of illness, organ failure and comorbidities on ICU admission				
APACHE II	28.0 (20.0;35.0)	25.0 (18.0;35.2)	29.0 (24.0;37.0)	<0.001*
SAPS II	54.0 (39.0;70.0)	49.0 (37.0;63.0)	61.0 (42.5;73.0)	<0.001*
TISS	50.0 (43.0;58.0)	47.0 (42.0;56.0)	53.0 (45.0;59.0)	<0.001*
SOFA	12.0 (9.0;15.0)	11.0 (9.0;14.0)*	13.0 (9.0;16.0)*	<0.001*
CCI	3.0 (2.0–5.0)*	3.0 (2.0–5.0)*	4.0 (3.0–6.0)*	<0.001*
Aetiology of ARDS				0.137
- Pneumonia	242 (54,7 %)	133 (55.0 %)	109 (45.0 %)	
- Immuninsufficiency	67 (15.1 %)	30 (44.8 %)	37 (55.2 %)	
- Acute on chronic	55 (12.4 %)	26 (47.3 %)	29 (52,7 %)	
- Trauma	29 (6,6 %)	20 (69.0 %)	9 (31.0 %)	
- Sepsis of extrapulmonary origin	27 (6.1 %)	17 (63.0 %)	10 (37.0 %)	
- Other	22 (5.0 %)	14 (63.3 %)	8 (36.7 %)	
Severity of lung failure (assessed on day1)				
AECC Definition				0.170
ALI	99 (22.4 %)	60 (60.6 %)	39 (39.4 %)	
ARDS	343 (77.6 %)	180 (52.5 %)	163 (47.5 %)	
Berlin Definition				0.149
Mild	99 (22.4 %)	60 (60.6 %)	39 (39.4 %)	
Moderate	210 (47.5 %)	117 (55.7 %)	93 (44.3 %)	
Severe	133 (30.1 %)	63 (47.4 %)	70 (52.6 %)	
PaO_2_/FiO_2_ [mmHg]	137 (92;193)	142 (95;199)	123 (89;178)	0.026*
Oxygenation index (OI)	16.9 (11.6;27.4)	16.4 (11.1;25.9)	18.5 (12.6;28.2)	0.063
Extracorporeal lung assist devices (ELAD)				<0.001*
No ELAD	186 (42.1 %)	130 (69.9 %)	56 (30.1 %)	
With ELAD	256 (57.9 %)	110 (43.0 %)	146 (57.0 %)	
- Only ECMO	146 (33.0 %)	61 (41.8 %)	85 (58.2 %)	
- Only ECLA	74 (16.7 %)	34 (46.0 %)	40 (54.0 %)	
- ECLA + ECMO	36 (8.14 %)	15 (41.7 %)	21 (58.3 %)	

Discrete variables are presented as number of percentage and were analysed with Chi square test for nonparametric samples. Continuous variables are presented as median and 25/75 percentiles and were analysed with Mann–Whitney-*U*-Test for nonparametric samples. * *p* < 0,05

*APACHE II* Acute Physiology And Chronic Health Evaluation II, *d* days, *ICU* intensive care unit, *LOS* length of stay, *SAPS II* Simplified Acute Physiology Score II, *SOFA* Sequential Organ Failure Assessment, *TISS* Therapeutic Intervention Scoring System, *CCI* Charlson comorbidity index, *F*_*i*_ *O*_*2*_ inspiratory fraction of oxygen, *iNO* inhalative nitric oxide, *PBW predicted body weight, P*_*a*_ *CO*_*2*_ arterial partial pressure of carbon dioxide, *P*_*a*_ *O*_*2*_ arterial partial pressure of oxygen, *PEEP* positive end-expiratory pressure, *P*_*mean*_ mean airway pressure, *P*_*peak*_ peak airway pressure, *V*_*t*_ tidal volume, *ECLA* extracorporeal lung assist, *ECMO* extracorporeal membrane oxygenation, *ELAD* extracorporeal lung assist devices. Complete data on hospital stay in referring institutions was available for 309 patients

On day 1 of the protocol, 99 patients (22.4 %) presented with a P_a_O_2_/F_i_O_2_ between 200 and 300, which corresponds to the stage of acute lung injury (ALI) in the AECC definition. Respectively, P_a_O_2_/F_i_O_2_ was below 200 in the remaining 343 patients (77.6 %). Applying the three stages of the Berlin definition, this corresponds to 99 patients (22.4 %) with mild, 210 patients (47.5 %) with moderate and 133 patients (30.1 %) with severe ARDS. The median for P_a_O_2_/F_i_O_2_ (*n* = 411) was 137 [93;193] and 16.9 [11.6;27.4] for OI (*n* = 391).

**Fig. 1:**
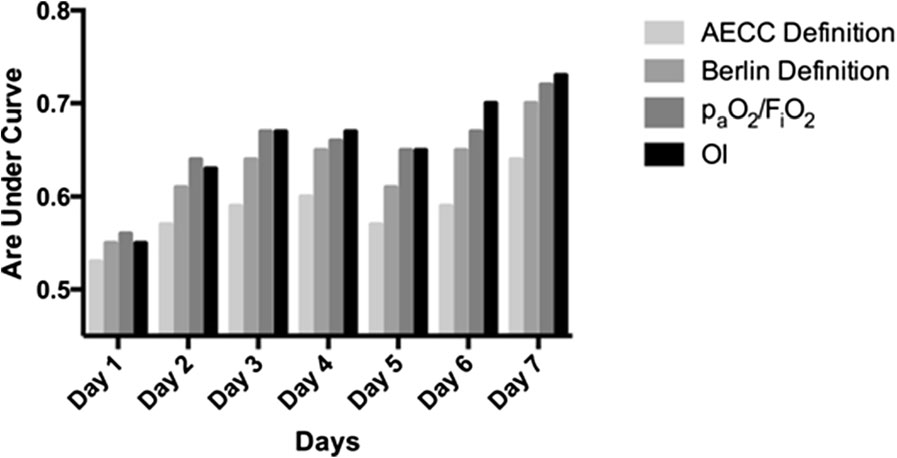
Predicitive validity for in-hospital mortality for the first seven days of ARDS after its diagnosis for AECC and Berlin Definition of ARDS, p_a_O_2_/F_i_O_2_, and oxygenation index. Area under ROC curve shown for the first seven days of ARDS by 4 categorizing options of severity of lung failure: AECC and Berlin Definition of ARDS, p_a_O_2_/F_i_O_2_ and OI

The predictive validity for in-hospital mortality of the four parameters mentioned above – AECC definition, Berlin definition, P_a_O_2_/ F_i_O_2_ and OI – was calculated for the first seven days on ICU (see Fig. 1). In general, the area under the curve was lowest for all four parameters on day 1 and highest on day 7. Given that we aimed to determine the earliest possible day for outcome prediction and that all parameters showed a monotonous increase until day 3, we decided to use clinical variables from that day for further analyses. As extracorporeal oxygenation was expected to have an impact on respiratory variables, sub-analyses were conducted regarding the predictive value of OI in patients with ELAD, without ELAD and in the entire patient population. As prognostic validity was highest in the group comprising all patients, we decided to evaluate all four categorizing variables regardless of possible extracorporeal oxygenation that might have been in place (see Additional file 4: Figure S1).

**Fig. 2:**
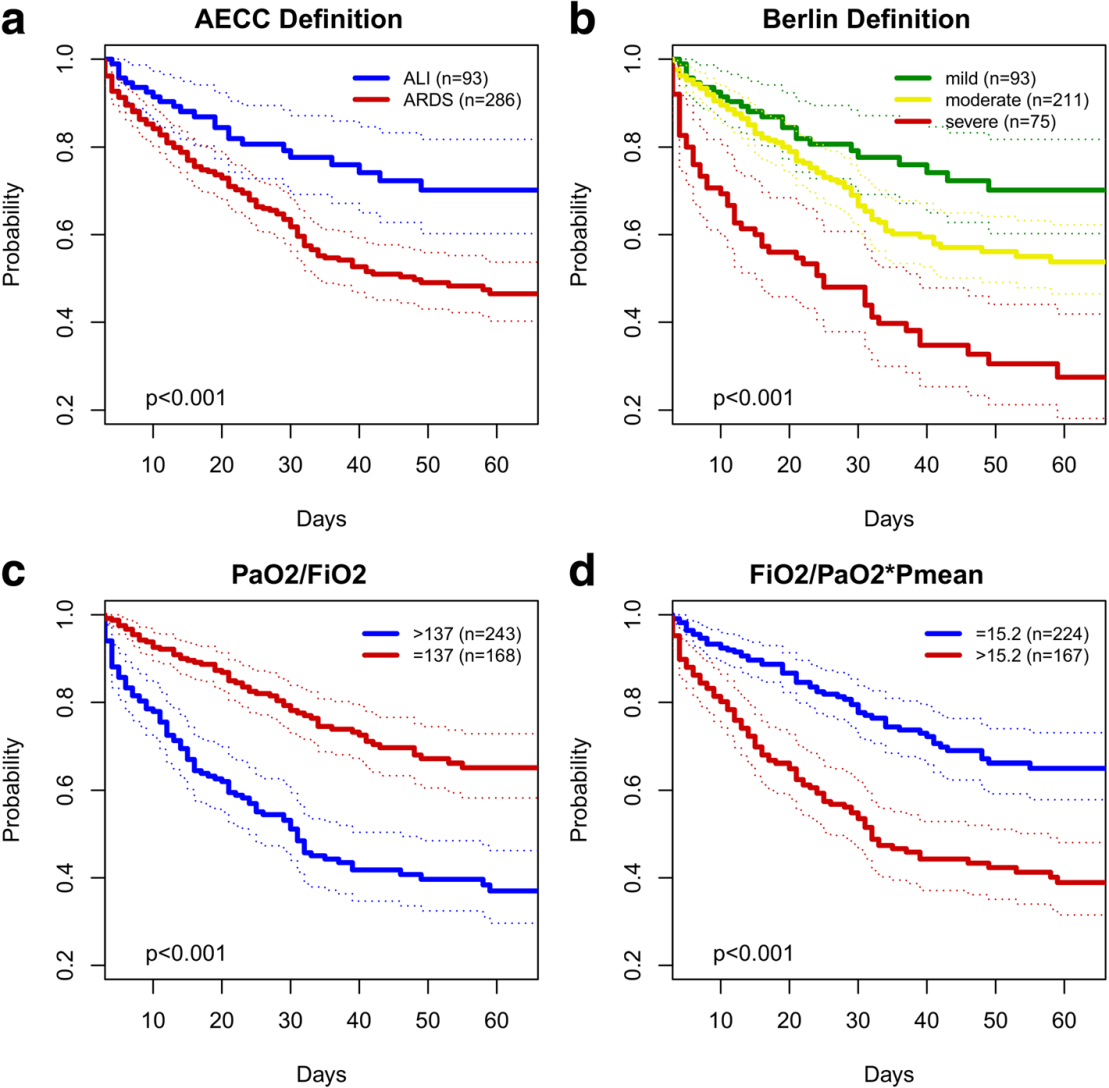
Survival curves for AECC and Berlin definition of acute respiratory distress syndrome, P_a_O_2_/F_i_O_2_ ratio and OI on day 3. Three hundred seventy nine patients had a P_a_O_2_/F_i_O_2_ ratio ≤ 300 mmHg on day 3 and have been grouped in the corresponding stages of the AECC (**a**) and Berlin definition (**b**). In total, P_a_O_2_/F_i_O_2_ was available for all 411 patients alive on day 3 (**c**). Values for F_i_O_2_/P_a_O_2_*P_mean_ were available for 391 patients being mechanically ventilated on that day (**d**). *AECC:* American-European Consensus Conference; *F*_*i*_ *O*_*2*_ *:* inspiratory fraction of oxygen; *F*_*i*_ *O*_*2*_ */P*_*a*_ *O*_*2*_ * P_*mean*_ describes oxygenation index*. P*_*a*_ *O*_*2*_ *:* arterial partial pressure of oxygen; *P*_*mean*_ *:* mean airway pressure, *OI*: oxygenation index

On day 3 of protocol, 31 patients had a PaO_2_/FiO_2_ > 300 mmHg. 32 patients died until day 3. Including data of all patients, resulting groups of all four classifications - AECC, Berlin definition, P_a_O_2_/F_i_O_2_ and OI – based on data from day 3 were depicted as Kaplan-Meier-curves in regards to in-hospital survival (Fig. 2). For the continuous parameters P_a_O_2_/F_i_O_2_ and OI, groups were identified by calculating the cut-off values (137 for P_a_O_2_/F_i_O_2_ and 15 for OI respectively) distinguishing between survival or death according to the Youden method described above. Resulting curves in each of the four Kaplan-Meier graphs were significantly different (p_Log rank_ < 0.001) from a univariate perspective. Multivariate regression analyses indicated that not a singular parameter may be considered for reliable mortality prediction (see Additional file 5: Table S2). Hence, stepwise backwards selection allowed the identification of clinically valid combinations of explanatory variables. In the resulting model, OI was the only one of the four investigated categorizing variables that remained significant (HR 1.03, 95 % CI 1.015–1.047, *p* < 0.001). With every one-point increase of OI, the risk of in-hospital death will increase by 3 %, whereas the risk of in-hospital death would increase by 36 % if the OI increased by 10 points. Use of extracorporeal lung assist devices did not prove to be an independent predictor (Table 2).

**Table 2:** Multivariate Cox regression on factors influencing hospital mortality

	*p* - value	HR	95 % CI
CCI	0.023*	1.08	1.010–1.149
OI on day 3 (per point)	<0.001*	1.03	1.015–1.047
Age (per year)	0.003*	1.02	1.006–1.029
TISS28 on admission	0.006*	1.02	1.007–1.043
C_rs_ on day 3	0.003*	0.98	0.971–0.994
Prone position on day 3	0.002*	0.81	0.711–0.928
BMI	0.097	0.98	0.956–1.004
pH on day 3	0.002*	0.03	0.002–0.275

Parameters considered in the multivariate regression model using backwards selection that had significant impact in univariate analyses: age, BMI, Charlson comorbidity index (CCI), ICU admission scores (APACHE II, SOFA, TISS28), ELAD (yes/no) as well as clinical variables that were assessed on day 3 (SOFA score), C_rs_ Compliance of respiratory system by C_rs_ = V_T_/ (P_plat_ - P_mean_) pH day 3, V_t_/PBW, P_peak_, Prone position, categories of Berlin definition and AECC definition, P_a_O_2_/F_i_O_2_. Data of 341 patients were considered. * *p* < 0,05. All parameters of the final regression model are presented, regardless of their significance

*BMI* Body Mass Index, *CCI* Charlson comorbidity index, *CI* confidence interval, *ELAD* extracorporeal lung assist devices (including extracorporeal lung assist (ECLA) and extracorporeal membrane oxygenation (ECMO), *F*_*i*_ *O*_*2*_ inspiratory fraction of oxygen, *HR* Hazard Ratio, *OI* oxygenation index, *PBW* predicted body weight, *P*_*peak*_ peak airway pressure, *TISS* Therapeutic Intervention Scoring System

OI – being an independent predictor in the final model of regression analysis – was then used to group patients concerning outcome criteria. Patients with an OI above the respective cut-off of 15 on day 3 had longer length of stay on ICU, longer length of hospital stay, and longer duration of mechanical ventilation. Furthermore, mortality was significantly higher, with patients less likely to be discharged to home or another facility (see Table 3).

**Table 3:** Clinical outcome parameters grouped by cut-off (15.2) of the oxygenation index F_i_O_2_/P_a_O_2_*P_mean_ (OI) on day 3

	All patients	*OI* day 3 < 15	*OI* day 3 ≥ 15	*p* - value
	*n* = 442	*n* = 242	*n* = 167	
Type of discharge				<0.001*
- Deceased	202 (45.7 %)	71 (29.3 %)	102 (61.1 %)	
- Discharged to home	60 (13.6 %)	40 (16.5 %)	17 (10.2 %)	
- Transfer to another hospital	95 (21.5 %)	54 (22.3 %)	27 (16.2 %)	
- Transfer to rehabilitation facility	85 (19.2 %)	59 (24.3 %)	21 (12.6 %)	
LOS ICU [d]	36.0 (25.0;57.0)	33.5, (21.8;53.0)	43.0 (31.0;61.0)	0.015*
LOS hospital [d]	43.0 (27.8;66.0)	41.0 (24.0;64.0)	48.0 (34.0;72.0)	0.030*
Duration of mechanical ventilation [h]	524 (320;862)	446 (274;772)	692 (497;1007)	<0.001*

Discrete variables are presented as median and percentage and were analysed with Chi square test for nonparametric samples. Continuous variables are presented as median and 25/75 percentiles and were analysed with Mann–Whitney-*U*-Test for nonparametric samples. * *p* < 0,05

*d* days, *h* hours, *ICU* intensive care unit, *n* number, *LOS* length of stay, *OI* oxygenation index calculated as median for each day by OI = (F_i_O_2_ / P_a_O_2_[mmHg]) × P_mean_ × 100). The cut-off value was derived from Youden – Index corresponding to the area under the ROC curve for OI on day 3 for all patients

## Discussion

In this study, we analysed early predictive values for mortality of the AECC- and Berlin definition of ARDS, P_a_O_2_/F_i_O_2_ and oxygenation index in critically ill ARDS patients at the reference centre of the Charité Berlin. We suggest that application of our standard operating procedures for ARDS treatment reduced or even eliminated the influence of previous, different treatment approaches. Among the four evaluated criteria to classify or quantify severity of ARDS, OI was found to be the most accurate parameter with respect to predictive validity.

Regarding the time of assessment, the third day after admission to our referral centre was found to represent the best compromise between earliness and accuracy of prognosis of mortality in this patient group. In the group of patients with an OI of 15 or greater on day 3, mortality was higher; length of stay (both in the ICU and hospital) and duration of mechanical ventilation were longer. Furthermore, non-survivors had a significantly longer length of stay and duration of mechanical ventilation in referring hospitals before being admitted to the ICU than survivors of ARDS.

The OI was originally designed as a predictive tool for pediatric patients with hypoxemic conditions [13, 14]. Later, this factor has been taken into consideration in adults suffering from ARDS [15, 16]. Our findings regarding compromised outcome in patients with an OI of 15 or greater are in line with previous findings indicating that the OI is equivalent to or even better than other mortality prediction parameters used for ARDS, and our study further substantiated this finding. We propose that the OI might be one of the preferable predictive parameters because it exclusively accounts for changes in mean airway pressure and thereby reflects invasiveness of mechanical ventilation to some extent. However, most large observational studies on predictive parameters in ARDS did not assess the OI to adjust the oxygenation ration ratio to the invasiveness of ventilation or did not report it [17, 18]. Moreover, currently used categorizing systems, i.e. the AECC and the Berlin definition of ARDS, do not consider the OI in their panels of defining variables [1, 3]. To include invasiveness of ventilation, a PEEP level of more or equal than five cm H_2_O became part of the definition. In contrast, the mean PEEP level in our study group was 17 cm H_2_O, which demonstrates the severity of illness in our patient population.

In contrast to OI, the P_a_O_2_/F_i_O_2_ ratio was not an independent predictor of mortality in our study. Although this parameter is often used to describe oxygenation status in critically ill patients, it also failed to predict clinical outcome at the onset of ARDS in recent studies [19, 20]. In this respect, our data support the concept that the P_a_O_2_/F_i_O_2_ ratio alone might not be suitable to determine clinical outcome in ARDS. One explanation might be that the P_a_O_2_/F_i_O_2_ ratio is a highly variable index depending on ventilator settings, conditions of patients and, to a more or lesser extend, routinely performed therapeutic interventions such as bronchoscopies or positioning. F_i_O_2_ by itself, independently of P_a_O_2_/F_i_O_2_, is usually not considered to select patients regarding their risk of poor outcome in larger clinical trails. However, it has been shown that after controlling for baseline P_a_O_2_/F_i_O_2_, F_i_O_2_ was able to predict mortality [21].

Villar and colleagues demonstrated that the P_a_O_2_/F_i_O_2_ ratio obtained 24 h after ARDS onset allowed a better risk classification when F_i_O_2_ was at least 0.5 with a PEEP of at least 10 cm H_2_O [22]. The OI was, however, not part of the Villar study. A side by side comparison in our study of the four different parameters on day 3 showed that the OI was the only parameter that was capable of predicting mortality in multivariate analyses. The other three, including the P_a_O_2_/F_i_O_2_ ratio, were not identified as independent predictors.

As a known fact, PEEP was not part of the AECC definition but was included in the Berlin definition for classifying ARDS. More studies were conducted to verify the predictive properties of PEEP in the definition of ARDS after the publications of AECC and Berlin guidelines. Since the Berlin definition defines the minimum of PEEP to be 5 cm H_2_O, many studies followed this assumption and concluded differing results. As an example, when Britos and coworkers categorized their cases as PEEP < 5 cm H_2_O (1.3 %) and ≥ 10 cm H_2_O (50 %), they found that after adjusting for P_a_O_2_/F_i_O_2_ baseline PEEP did not predict mortality [21]. In contrast, in our study the median PEEP was 17 cm H_2_O, which may be one of the conditions facilitating the finding of a predictable parameter when requiring a higher PEEP. Goligher and coworkers observed in a patient group with a median PEEP of 9.5 cm H_2_O, that positive oxygenation response to PEEP elevation may predict mortality [23]. Golighers conclusions may not be applied to our patient population that was treated with median PEEP of 17 cm H_2_O. In this regard, the use of a PEEP level of 5 cm H_2_O recommended by the Berlin definition might not be appropriate to discriminate the severity of illness and does not allow reliable mortality prediction, respectively. Furthermore, if a PEEP level of 5 cm H_2_O would have been applied in our patient population, it is strongly expected that the number of severe ARDS cases would increase. Hence, the number of severe cases reported in this manuscript is supposedly underestimated from this perspective. In line with aforementioned studies, this observation underlies the importance of standardized conditions for ARDS classification in order to render investigations from different settings comparable.

In our study, the third day of P_a_O_2_/F_i_O_2_ < 300 mmHg in our centre was found to represent the best compromise between earliness and accuracy of prognosis of mortality in this patient group. As a special referral centre we are sometimes unable to identify the exact onset of ARDS, which is a limitation of our study. We suggest that the very different treatment approaches in pre-treating hospitals bias comparability of patients at the time of referral to our centre. In the study by Peek and colleagues, the different treatment procedures in hospitals other than ECMO centres were considered as a major limitation when comparing patients [24]. In our opinion, algorithm-guided –and most importantly – homogeneous standard treatment approaches including ventilator settings, inhalation of nitric oxide, prone positioning or volume therapy in an ARDS centre may eliminate these confounding effects. In this context, our data suggests that length of stay in hospitals before transfer to a specialized ARDS treatment centre could impact mortality.

## Conclusions

The Berlin definition has marked a major step for a uniform classification of ARDS. As major finding of our study, the OI has been proven to be a more suitable parameter to predict mortality of patients suffering from ARDS in national referral centres when compared to P_a_O_2_/F_i_O_2_ ratio, the AECC or Berlin definition. The OI appears to be especially helpful for prediction of mortality on day three after admission to a specialized referral centre, as a response to standardised ARDS treatment may be observed by that time. Secondly, early patient transfers to specialized ARDS centres are associated with increased survival.

## Additional files

Additional file 1: Table S1. Selected patient-specific parameters on day 1 and day 3. (DOC 49 kb)
Additional file 2: Table S3. Number of patients in prone position on each day. IQR: 1d-2d. (DOC 25 kb)
Additional file 3: Table S4. Number of patients receiving extracorporeal lung assist devices (ELAD) on each day. (DOC 26 kb)
Additional file 4: Figure S1. Predictive validity of oxygenation index for in-hospital mortality for the first seven days of ARDS grouped by need of extracorporeal lung assist devices (ELAD). (JPG 38 kb)
Additional file 5: Table S2. Full model of multivariate Cox regression analysis on factors influencing hospital mortality. (DOC 37 kb)

## Abbreviations

AECC: American European consensus conference on ARDS
ALI: Acute lung injury
APACHE II: Acute physiology and chronic health evaluation II
ARDS: Acute respiratory distress syndrome
AUC: Area under curve
CCI: Charlson comorbidity index
COPD: Chronic obstructive pulmonary disease
ECLA: Extracorporeal lung assist
ECMO: Extracorporeal membrane oxygenation
ELAD: Extracorporeal lung assistant devices
F_i_O_2_: Fraction of inspired oxygen
ICU: Intensive care unit
OI: Oxygenation index
P_a_O_2_: Arterial partial pressure of oxygen
PBW: Predicted body weight
PEEP: Positive endexspiratory pressure
SAPS II: Simplified acute physiology score
SOFA: Sequential organ failure assessment
V_T_: Tidal volume
