# Supplementary material for: Predictors of survival in critically ill patients with acute respiratory distress syndrome (ARDS): an observational study
Source: BMC Anesthesiol. 2016 Nov 8;16:108. doi: 10.1186/s12871-016-0272-4 (PMC5100178; doi:10.1186/s12871-016-0272-4)
Supplement: Additional file 1: Table S1. — Selected patient-specific parameters on day 1 and day 3. (DOC 49 kb) [file 12871_2016_272_MOESM1_ESM.doc]

Additional file 1: Table S1. Selected patient-specific parameters on day 1 and day 3

|  | **All patients**  **n = 442** | **Survivor**  **n = 240** | **Non-survivor**  **n = 202** | **p - value** |
| --- | --- | --- | --- | --- |
| **Day 1** |  |  |  |  |
| SOFA score | 12 (9;15) | 11 (9;14)* | 13 (9;16)* | < 0.001* |
| F_i_O_2_ | 88.2 (66.0;99.5) | 83.0 (65.0;99.0) | 92.8 (70.0;100) | 0.066 |
| PEEP [cmH_2_O ] | 17.3 (15.3;20.4) | 17.3 (15.3;20.4)* | 16.3 (14.5;20.0)* | 0.014* |
| P_mean_ [cmH_2_O ] | 24.5 (21.4;28.1) | 24.5 (22.2;28.0) | 24.5 (21.4;28.2) | 0.560 |
| P_peak_ [cmH_2_O] | 35.7 (31.9;38.8) | 34.9 (31.6;38.5)* | 36.2(32.6;39.8)* | 0.017* |
| V_t_ [ml] | 388 (284;496) | 430 (328;518)* | 350 (244;452)* | < 0.001* |
| V_t_/PBW  [ml/kg PBW] | 5.95 (4.58;7.41) | 6.37 (5.06;7.68)* | 5.50 (3.96;7.02)* | < 0.001* |
| Compliance [ml/ cmH_2_O ] | 27.8 [19.3;40.1] | 33.5 [24.2;46.1] | 23.3 [15.0;31.4] | < 0.001* |
| P_a_O_2_ [mmHg) | 97.1 (75.7;130) | 100 (79.9;135)* | 91.3 (71.6;123)* | 0.021* |
| P_a_CO_2_ [mmHg] | 52.6 (42.7;63.4) | 49.5 (40.9;57.9)* | 56.5 (44.7;66.1)* | < 0.001* |
| pH | 7.33 (7.26;7.41) | 7.35 (7.29;7.41)* | 7.31 (7.22;7.39)* | < 0.001* |
| iNO | 329 (74.4%) | 169 (51.4%) | 160 (48.6%) | 0.063 |
| **Day 3** |  |  |  |  |
| SOFA score | 13 (10;16) | 12 (9;15) | 13 (11;16) | 0.008 |
| F_i_O_2_ | 63.2 (47.5;81.0) | 59.0 (45.0;75.0)* | 71.5 (51.0;91.1)* | < 0.001* |
| PEEP [mbar] | 16.3 (14.3;19.8) | 16.4 (14.3;19.8) | 16.3(14.5;19.4) | 0.943 |
| P_mean_ [mbar] | 22.4 (19.4;24.9) | 22.4 (19.4;24.5) | 22.5 (19.4;25.5) | 0.114 |
| P_peak_ [mbar] | 32.2 (29.2;34.8) | 31.9 (28.8;34.2)* | 32.6 (29.4;35.9)* | 0.011* |
| V_t_ [ml] | 348 (193;451) | 386 (243;481)* | 282 (159;415)* | < 0.001* |
| V_t_/PBW  [ml/kg PBW] | 5.33 (2.92;6.83) | 5.99 (4.00;7.22)* | 4.39 (2.57;6.30)* | < 0.001* |
| Compliance  [ml/ cmH_2_O ] | 29.4 [19.4;41.7] | 33.6 [24.3;48.7] | 23.2 [14.9;33.5] | <0.001* |
| P_a_O_2_ [mmHg) | 90.6 (75.3;112) | 95.8 (80.5;116)* | 81.4 (69.5;104)* | < 0.001* |
| P_a_CO_2_ [mmHg] | 45.8 (40.5;53.8) | 46.2 (41.3;53.8) | 45.7 (40.2.53.9) | 0.520 |
| pH | 7.38 (7.33;7.43) | 7.39 (7.34;7.44)* | 7.38 (7.31;7.42)* | 0.016* |
| iNO | 353 (75.8%) | 182 (75.8%) | 171 (84.7%) | 0.029* |

Discrete variables are presented as median and percentage and were analysed with Chi square test for nonparametric samples. Continuous variables are presented as median and 25/75 percentiles and were analysed with Mann-Whitney-U-Test for nonparametric samples. * p < 0,05. *Compliance:* of respiratory system calculated by V_T_/ (P_plat_ - P_mean_); *F_i_O_2_:* inspiratory fraction of oxygen; *iNO:* inhalative nitric oxide; *PBW: predicted body weight; P_a_CO_2_:* arterial partial pressure of carbon dioxide; *P_a_O_2_:* arterial partial pressure of oxygen; *PEEP:* positive end-expiratory pressure; *P_mean_:* mean airway pressure; *P_peak_:* peak airway pressure; *SOFA:* sequential organ failure assessment; *V_T_:* tidal volume
