# Supplementary material for: Predictors of survival in critically ill patients with acute respiratory distress syndrome (ARDS): an observational study
Source: BMC Anesthesiol. 2016 Nov 8;16:108. doi: 10.1186/s12871-016-0272-4 (PMC5100178; doi:10.1186/s12871-016-0272-4)
Supplement: Additional file 2: Table S3. — Number of patients in prone position on each day. IQR: 1d-2d. (DOC 25 kb) [file 12871_2016_272_MOESM2_ESM.doc]

Additional file 2: **Table S3**. Number of patients in prone position on each day. IQR: 1d-2d.

|  | Day 1 | Day 2 | Day 3 | Day 4 | Day 5 | Day 6 | Day 7 | > Day 7 |
| --- | --- | --- | --- | --- | --- | --- | --- | --- |
| n | 179 | 134 | 22 | 8 | 6 | 1 | 3 | 27 |
