# Supplementary material for: Predictors of survival in critically ill patients with acute respiratory distress syndrome (ARDS): an observational study
Source: BMC Anesthesiol. 2016 Nov 8;16:108. doi: 10.1186/s12871-016-0272-4 (PMC5100178; doi:10.1186/s12871-016-0272-4)
Supplement: Additional file 3: Table S4. — Number of patients receiving extracorporeal lung assist devices (ELAD) on each day. (DOC 26 kb) [file 12871_2016_272_MOESM3_ESM.doc]

Additional file 3: **Table S4**. Number of patients receiving extracorporeal lung assist devices (ELAD) on each day

|  | Day 1 | Day 2 | Day 3 | Day 4 | Day 5 | Day 6 | Day 7 | > Day 7 |
| --- | --- | --- | --- | --- | --- | --- | --- | --- |
| n | 142 | 63 | 19 | 8 | 5 | 4 | 2 | 13 |
