# Supplementary figures and images for: Predictors of survival in critically ill patients with acute respiratory distress syndrome (ARDS): an observational study
Source: BMC Anesthesiol. 2016 Nov 8;16:108. doi: 10.1186/s12871-016-0272-4 (PMC5100178; doi:10.1186/s12871-016-0272-4)

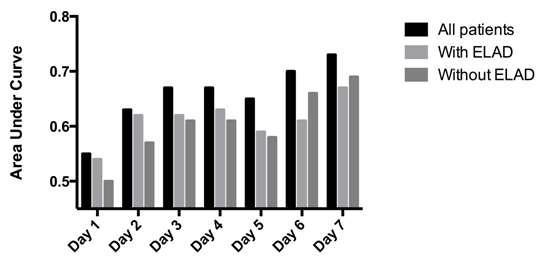

Supplement: Additional file 4: Figure S1. — Predictive validity of oxygenation index for in-hospital mortality for the first seven days of ARDS grouped by need of extracorporeal lung assist devices (ELAD). (JPG 38 kb) [file 12871_2016_272_MOESM4_ESM.jpg]
