# Supplementary material for: Predictors of survival in critically ill patients with acute respiratory distress syndrome (ARDS): an observational study
Source: BMC Anesthesiol. 2016 Nov 8;16:108. doi: 10.1186/s12871-016-0272-4 (PMC5100178; doi:10.1186/s12871-016-0272-4)
Supplement: Additional file 5: Table S2. — Full model of multivariate Cox regression analysis on factors influencing hospital mortality. (DOC 37 kb) [file 12871_2016_272_MOESM5_ESM.doc]

Additional file 5: **Table S2**. Full model of multivariate Cox regression analysis on factors influencing hospital mortality

|  | **p – value** | **HR** | **95% CI** |
| --- | --- | --- | --- |
| pH on day 3 | <0.001 | 0.009 | 0.001-0.115 |
| CCI | 0.014 | 1.087 | 1.017-1.161 |
| TISS score on ICU admission | 0.017 | 1.022 | 1.004-1.041 |
| Prone position on day 3 | 0.018 | 0.840 | 0.726-0.971 |
| C_rs_ on day 3 | 0.019 | 0.977 | 0.958-0.996 |
| Age [y] | 0.117 | 1.010 | 0.997-1.023 |
| SOFA score on day 3 | 0.173 | 0.957 | 0.899-1.019 |
| APACHE II on ICU admission | 0.222 | 1.017 | 0.990-1.044 |
| P_a_O_2_/F_i_O_2_ | 0.284 | 0.996 | 0.990-1.003 |
| F_i_O_2_/P_a_O_2_*Pmean (OI) | 0.284 | 1.019 | 0.984-1.055 |
| Extracorportal oxygenation | 0.328 | 1.280 | 0.781-2.098 |
| Body Mass Index | 0.359 | 0.987 | 0.961-1.014 |
| AECC Definition | 0.364 | 0.590 | 0.189-1.845 |
| TV / PBW on day 3 | 0.424 | 1.053 | 0.928-1.194 |
| P_peak_ on day 3 | 0.424 | 0.979 | 0.929-1.031 |
| SOFA score on ICU admission | 0.945 | 0.997 | 0.921-1.080 |
| Berlin Definition | 0.970 | 0.973 | 0.241-3.933 |

Inline with the assumption that there is no singular parameter that may be considered for outcome prediction in ARDS patients, none of the four ARDS classifications (AECC / Berlin definition, P_a_O_2_/F_i_O_2_, OI) was statistically significant before stepwise variable selection.

*BMI:* Body Mass Index; *CCI:* Charlson comorbidity index; *CI:* confidence interval; *C_rs_:* Compliance of the respiratory system; *FiO_2_:* inspiratory fraction of oxygen; *HR:* Hazard Ratio; *OI:* oxygenation index; *PBW:* predicted body weight; *P_peak_:* peak airway pressure; *TISS:* Therapeutic Intervention Scoring System
